# Gut lymph purification alleviates acute lung injury induced by intestinal ischemia-reperfusion in rats by removing danger-associated molecular patterns from gut lymph

**DOI:** 10.1016/j.heliyon.2024.e25711

**Published:** 2024-02-02

**Authors:** Wei Zhang, Can Jin, Shucheng Zhang, Linlin Wu, Bohan Li, Meimei Shi

**Affiliations:** a Department of Critical Care Medicine, The People's Hospital of Leshan, Leshan City, Sichuan Province, 614008, China; b Department of Critical Care Medicine, Affiliated Hospital of Zunyi Medical University, Zunyi, Guizhou, China; c Zunyi Medical University, Zunyi, Guizhou, China; d Key Laboratory of Resource Biology and Biotechnology in Western China, Ministry of Education. School of Medicine, Northwest University. Xi'an, 710069, Shanxi, China

**Keywords:** Danger-associated molecular patterns, Gut lymph, Purification, Ischemia-reperfusion injury, Acute lung injury

## Abstract

**Background:**

The potential effect of removing danger-associated molecular patterns (DAMPs) from gut lymph on reducing acute lung injury (ALI) induced by gut ischemia-reperfusion injury (GIRI) is uncertain. This study aimed to investigate whether gut lymph purification (GLP) could improve GIRI-induced acute lung injury in rats by clearing danger-associated molecular patterns.

**Materials and methods:**

Rats were divided into four groups: Sham, GIRI, GIRI + gut lymph drainage (GLD), and GIRI + GLP. After successful modeling, lung tissue samples were collected from rats for hematoxylin-eosin (HE) staining and detection of apoptotic indexes. We detected the DAMPs levels in blood and lymph samples. We observed the microstructure of AEC Ⅱ and measured the expression levels of apoptosis indexes.

**Results:**

The GIRI group showed destruction of alveolar structure, thickened alveolar walls, and inflammatory cell infiltration. This was accompanied by significantly increased levels of high mobility group protein-1 (HMGB-1) and Interleukin-6 (IL-6), while reduced levels of heat shock protein 70 (HSP 70) and Interleukin-10 (IL-10) in both lymph and serum. In contrast, the lung tissue damage in the GIRI + GLP group was significantly improved compared to the GIRI group. This was evidenced by a reduction in the expression levels of HMGB-1 and IL-6 in both lymph and serum and an increase in HSP 70 and IL-10 levels. Additionally, organelle structure of AEC II was significantly improved in the GIRI + GLP group compared to the GIRI group.

**Conclusions:**

GLP inhibits inflammation and cell apoptosis in GIRI-induced ALI by blocking the link between DAMPs and mononuclear phagocytes, reducing the severity of ALI.

## 1 Introduction

During the acute phase reaction of critical illness, blood flow redistribution occurs as a protective mechanism. However, this response results in gut ischemia-reperfusion injury (GIRI) [1]. In the 1980s, the gut was proposed to play a central role in the trigger theory of sepsis and multiple organ dysfunction syndrome (MODS) [2]. The gut lymph theory was first proposed by Deitch et al., suggesting that after GIRI, the intestinal mucosal barrier is compromised, and intestinal cells that have undergone programmed lytic necrosis release danger-associated molecular patterns (DAMPs) into the intestinal mucosal tissue space [3]. DAMPs are absorbed into the gut lymph fluid of the gut lymph system and subsequently diffuse into the systemic blood circulation, activating the mononuclear phagocyte system. This process creates a continuous cycle that contributes to the cascade of inflammation [[4], [5], [6]].

The gut lymph theory is the main underlying mechanism of acute lung injury (ALI) caused by GIRI. DAMPs carried in the gut lymph enter the systemic blood circulation through the pulmonary vascular bed and activate mononuclear phagocytes, triggering an inflammatory cascade effect [7,8]. Recent experimental evidence has shown that gut lymph serves as a carrier of DAMPs, leading to ALI and MODS in both the lungs and systemic blood circulation [1]. Some studies showed that gut lymph drainage (GLD) and gut lymph ligation can significantly reduce the risk of organ injury in rats [[9], [10], [11], [12], [13], [14]], suggesting that the gut lymph fluids play an important role in inflammation and MODS [[15], [16], [17], [18], [19]]. However, these approaches are not practical for clinical use [20,21]. Therefore, we proposed a therapeutic measure known as the gut lymph purification (GLP) technique, which aims to disrupt the critical link between DAMPs and mononuclear phagocytes to mitigate inflammation and prevent MODS.

According to the gut lymph theory, GLP therapy refers to a therapeutic technique of establishing an extracorporeal circulation pathway of gut lymph through gut lymph vessel puncture and catheterization, then draining the gut lymph liquids out of the body, and then clearing the pathogenic factors (DAMPs) in gut lymph through a special purification device before being infusion back into the body. The key to the success of the GLP lies in the filtration membrane. oXiris (Gambro), which is an adsorbed hemofiltration membrane based on the hydrogel structure of a propylene and sodium mesylate polymer (AN69), has been widely used to treat sepsis and septic shock in recent years [22,23]. We aimed to investigate whether the oXiris-based GLP can reduce the injury of distal organs, such as the lungs, by removing DAMPs from the gut lymph fluid after GIRI-induced ALI.

## 2 Materials and methods

The research was conducted under the ARRIVE 2.0 guideline (www.arriveguidelines.org).

### 2.1 Animals

All animal experiments were carried out following the guidance of the Animal Care and Use of Laboratory Animals and were approved by the Ethics Committee of Experimental Animals and Use of Laboratory Animals of Zunyi Medical University. Healthy adult Sprague-Dawley rats (specific-pathogen-free grade) aged 20–22 weeks and weighing 250 ± 50 g were purchased from Hunan Slack Jingda Experimental Animal Co., Ltd., with the license number SCXK (Xiang) 2019-0004. All rats had free access to the same water and chow (Xiaohe Technology Development Company, Pizhou City, Jiangsu Province, China). The animals were housed under controlled conditions at 22 ± 2 °C and 45–55 % of humidity in a 12-h light/dark cycle.

### 2.2 Grouping and modeling

Those health adult rats were randomly divided into four groups: (i) Sham group (n = 12), which underwent gut lymph stem puncture and catheterization (as described in *the Jugular venipuncture catheterization section*) without clamping the superior mesenteric artery (SMA) and collection of gut lymph fluid for testing with no other interventions; (ii) GIRI [24] group (n = 12), which underwent the same procedure as the Sham group but with SMA clamped for 60 min, followed by loosening for another 120 min; (iii) GIRI + GLD group (n = 12), which underwent the same procedure as the GIRI group, but the collected gut lymph fluid was infused back into the body through the jugular vein; (iv) GIRI + GLP group (n = 12), which underwent the same procedure as the GIRI + GLD group, but the collected gut lymph fluid was treated with the oXiris GLP system before being infused into the body. Those rats were anesthetized with 30 mg/kg 1 % pentobarbital sodium via intraperitoneal injection and humanely euthanized under deep anesthesia.

### 2.3 Adsorption column based on oXiris biofilm

The oXiris adsorption column was fabricated: the coats were sawed neatly along a 0.25 mL scale using two 1 mL injectors with the core rod removed. The rubber chips adhering to the coats were washed with saline solution and dried. After combining the broken ends of both coats, the oXiris filter wires were filled in. The joint of the coats of the two syringes was pasted across using a pre-cut 1.5 cm thick disposable medical transparent dressing for compactness. The oXiris adsorption column was sterilized with ethylene oxide, sealed, and stored at room temperature.

### 2.4 Jugular venipuncture catheterization for re-infusion of gut lymph fluid

Jugular venipuncture catheterization was performed: after establishing the GLD model, the skin from the right sternoclavicular connection at the midpoint of the neck was vertically incised to 1.5 cm to expose the subcutaneous connective tissue. The jugular vein was visualized and isolated by gradual blunt separation. To induce congestion and swelling, the proximal and distal veins by clamped with a noninvasive vascular clamp. A small aperture was cut in the middle of this section, which was slightly smaller than the external diameter of the silica gel catheter that was pre-flushed with heparin saline. The vascular clamp was then loosened, and the silica gel catheter was sutured and fixed within the vein orifice. Finally, the connective tissue and skin were sutured. To perform gut lymph fluid reperfusion, the tubing of the peristaltic pump was filled with saline in advance and connected to the jugular vein of the rats.

### 2.5 Cell separation and extraction

Monocyte separation was carried out: 5 mL of whole blood from the modeled mouse was diluted with 5 mL of phosphate buffered solution (PBS). The diluted blood was dropped onto the surface of the separation solution and then centrifuged at 2000 rpm for 20 min. The middle buffy coat cells were collected into a clean centrifuge tube and washed with PBS. After centrifugation, the supernatant was discarded, and the cell suspension was added to a prepared culture plate. To induce macrophages and dendritic cells, 50 ng/mL of phorbol-12-myristate-13-acetate (PMA) was added to the isolated mononuclear cell culture medium, and the cells were cultured for 48 h.

Lymphocyte separation was performed by collecting the lymph fluid and centrifuging it at 1500 rpm for 3 min. The supernatant was then discarded, and the medium was added to resuspend the cells. The cell suspension was added to a culture dish for further culture.

To separate alveolar epithelial cells, rat lung tissue was collected and added to a buffer solution containing 10 % bi-antibody. 0.25 % pancreatin was injected for digestion at 37 °C for 25 min. The lung tissues were slightly cut, and the complete medium was added to end the digestion. All the liquid was collected, filtered with a 70 μm filter, and centrifuged at 1500 rpm for 5 min to collect the cells. The cells were then resuspended and added to 500 μg/mL IgG-coated Petri dishes for culture and purify for half an hour. The purified cell suspension was collected and added to a prepared culture dish. A mark was made, and the cells were placed in an incubator for normal culture.

### 2.6 Histological examination

The collected lung tissue was fixed with 4 % paraformaldehyde for 24 h at room temperature. It was then embedded in paraffin and dehydrated before being cut into thin slices with a thickness of 5 μm. The sections were stained with hematoxylin for 5 min and 0.5 % eosin for 3 min at room temperature. The tissue section was observed under an optical microscope (CX41, OLYMPUS).

### 2.7 Detection of DAMPs in the gut lymph fluid and plasma

DAMPs in the gut lymph fluid and plasma were detected using enzyme-linked immunosorbent assay (ELISA), following the manufacturer's instructions.

### 2.8 Western blot analysis

Sample was lysed with 400 μL of lysis buffer (P0013, Beyotime Institute of Biotechnology) containing 100 mmol/L phenylmethylsulphonyl fluoride (PMSF) (ST506, Beyotime Institute of Biotechnology) for 30 min, and then transferred to an Eppendorf tube. The lysate was then centrifuged for 10 min at 2000 r/min. The protein concentration was determined using the bicinchoninic acid kit. Protein was denatured, loaded, and subjected to sodium dodecylbenzene sulfonate gel electrophoresis for 2 h and then transferred to the membrane with a constant current of 300 mA for 80 min. The membrane was then incubated with the primary antibody at 4 °C overnight, followed by incubation with the secondary antibody at room temperature for 2 h. The ECL luminescent liquid was dropped on the film, which was then exposed in the gel imaging system (Chemi DocTM XRS+, Bo Le Biomedical Products (Shanghai) Co., Ltd.). The gray value of each band was analyzed using "ImageJ" software.

### 2.9 Quantitative real-time polymerase chain reaction (qRT-PCR)

Total RNA was isolated using TRIzol. The concentration and purity of RNA (OD260/OD280) were measured using a UV–Visible spectrophotometer. cDNA was synthesized using an RNA reverse transcription kit. The mRNA expression was quantified using a qRT-PCR instrument (CFX Connect™ Real Time, Bole Life Medical Products (Shanghai) Co., Ltd.). The internal control β-actin F was used to normalize the expression levels. Relative expression levels of bcl-2, Bax, FAS, and FASL were calculated using the 2^-△△Ct^ method [25]. The primers for the target genes were provided by General Biosystems (Anhui) Co., Ltd. The primer sequences were as follows: bcl-2, forward 5′-GCGTCAACAGGGAGATGTCA-3′ and reverse 5′ TTCCACAAAGGCATCCCAGC-3'; Bax, forward 5′-GCGATGAACTGGACAACAAC-3′ and reverse 5′ GCAAAGTAGAAAAGGGCAACC-3'; Fas, forward 5′-GCCCATTTTGCTGTCAACCG-3′ and reverse 5′ GTCTTCAAGTCCACACGAGGT-3'; Fas-L, forward 5′-CACCAACCACAGCCTTAGAGT-3′and reverse 5′ GAGCGGGGGTTCCCTGTTAAG-3'; β-actin, forward 5′-GCCATGTACGTAGCCATCCA-3′ and reverse 5′ GAACCGCTCATTGCCGATAG-3'.

### 2.10 Detection of the function of mononuclear phagocytes

Mononuclear phagocytes were co-cultured with lymphocytes that were isolated from gut lymph fluid, and the status and function of the mononuclear phagocytes were evaluated. The proliferation rate was measured using the cell counting kit-8 **(**CCK8). The apoptosis rate was measured using flow cytometry (Novocyte 2060R, Aisen Biology (Hangzhou) Co., Ltd.). The inflammatory factor secretion was measured using ELISA.

### 2.11 Detection of the dendritic cells’ antigen-presenting function

The dendritic cell concentration was adjusted to 5 × 10^4^/mL. Then, 1.5 mL of the dendritic cell suspension was added to each well of a 6-well plate. Subsequently, 150 μL of gut lymph fluid was added to each well and incubated in a 5 % CO_2_ incubator at 37 °C for 24 h. Afterward, the cells were scraped, suspended with 5 mL of PBS, and centrifuged at 250 g for 10 min. The pellet was resuspended in 5 mL of PBS and centrifuged again for 10 min. Cells in PBS were resuspended, and 1 μL of OX-6 and 2 μL of OX-42 were added to the suspension. The sample was protected from light for up-flow detection within 1 h.

### 2.12 Transmission electron microscopy (TEM)

Type II alveolar epithelial cells (AEC II) were identified using TEM. After incubating AEC II cells for 48 h, the cells were digested with 0.125 % trypsin. The resulting cell suspension was centrifuged at 100×*g* at 4 °C for 10 min. The supernatant was removed, and the cells were fixed with 4 % glutaraldehyde for 24 h at room temperature. Cell pellets were rinsed three times for 10 min at 4 °C in PBS and then fixed with 1 % osmium tetroxide at 4 °C for 30 min. The pellets were washed again three times with PBS and observed using a JEOL JEM-1230 (80 KV) TEM.

### 2.13 Statistical analysis

Statistical analysis was carried out using IBM SPSS software version 25.0. The data were expressed as mean ± standard deviation. To compare the differences between groups, a one-way analysis of variance followed by post hoc Dunnett's T-test was performed. A p-value less than 0.05 was considered statistically significant.

## 3 Results

### 3.1 GIRI induces ALI in rats

**Fig. 1:**
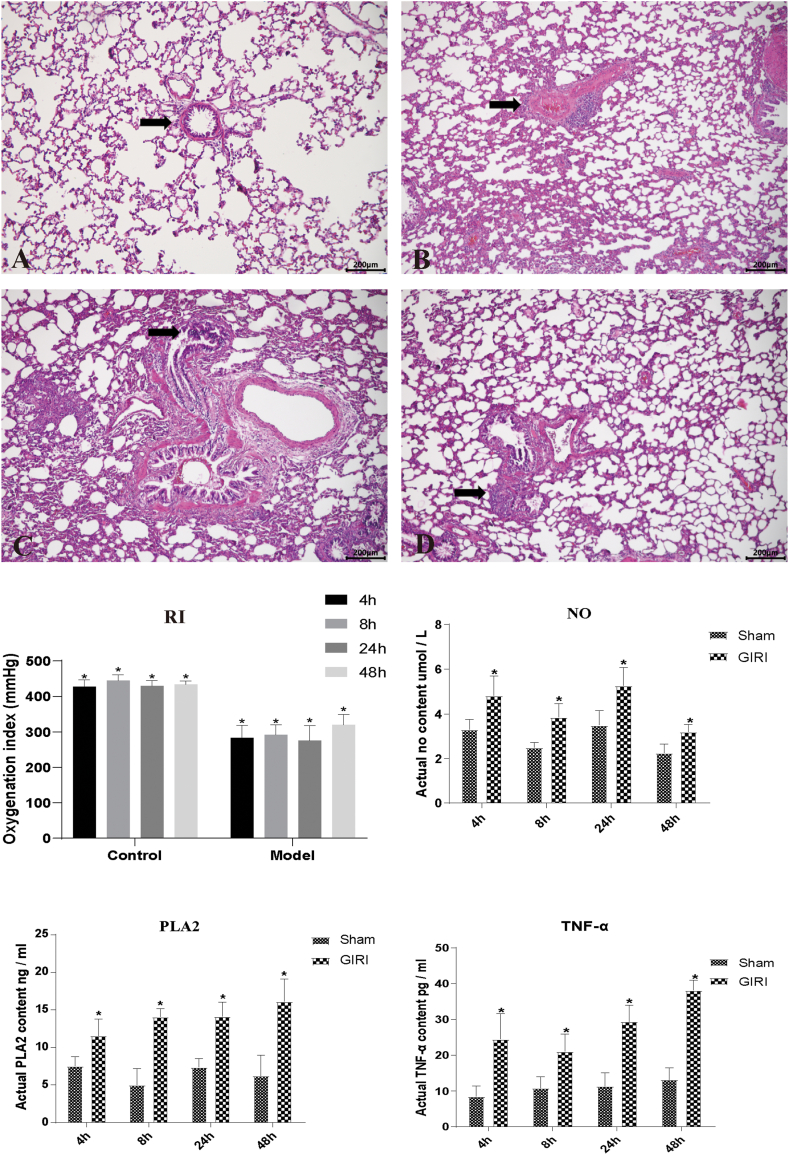
Gut ischemia-reperfusion injury (GIRI) induced acute lung injury (ALI) in Rats. The rats in the Sham group underwent intestinal manipulation only without ligation of the superior mesenteric artery (SMA), whereas the GIRI group underwent SMA ligation for 1 h followed by reperfusion. (A–D) Pathological changes were evaluated using hematoxylin and eosin (H&E) staining and light microscopy at 4 (A), 8 (B), 24 (C), and 48 (D) hours after intestinal ischemia. The black arrow highlights the changes in alveoli over time, with panel A showing alveolar wall thickening and a small amount of inflammatory cell infiltration, panel B demonstrating pulmonary alveolar edema and congestion, panel C indicating significant inflammatory cell infiltration and alveolar wall destruction in the alveolar cavity, and panel D exhibiting severe alveolar edema with the disappearance of the alveolar wall. Scale bar, 200 μm. (E) The respiratory index (RI) was measured to assess respiratory function. (F–H) Enzyme-linked immunosorbent assay (ELISA) was used to measure the serum levels of nitric oxide (NO), lipoprotein-associated phospholipase A (PLA2), and tumor necrosis factor-α (TNF-α). Data are expressed as mean ± standard deviation (SD). *P < 0.05 vs. sham; n = 6. GIRI, gut ischemia-reperfusion injury; RI, respiratory index; NO, nitric oxide; PLA2, lipoprotein-associated phospholipase A; TNF, tumor necrosis factor.

To validate the success of the GIRI-induced ALI model, we evaluated the pathological changes in alveolar tissue, oxygenation index (OI), and serum markers. The results of hematoxylin and eosin staining showed that the alveolar tissue from the Sham group appeared normal, while the alveolar structure in the GIRI group was evidently damaged (Fig. 1A–D) at 4, 8, 24, and 48 h after modeling. There was a significant reduction in OI value after modeling (Fig. 1E). ELISA results showed that the levels of nitric oxide (NO), phospholipase A2 (PLA2), and tumor necrosis factor-α (TNF-α) in the serum of GIRI rats increased at all time points. However, the levels of these indices were significantly higher at 24 h, leading us to select this time point for subsequent experiments (Fig. 1F–H).

### 3.2 GLP alleviates GIRI-induced ALI in rats

**Fig. 2:**
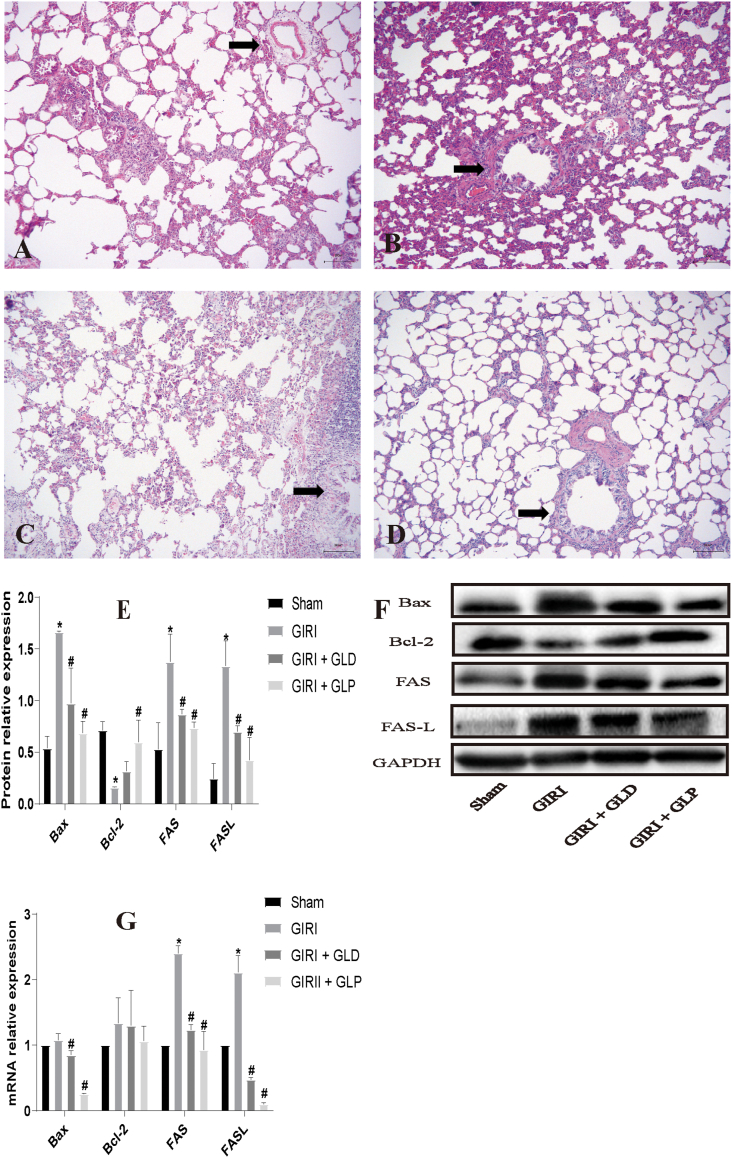
Gut lymph purification (GLP) alleviated GIRI-induced ALI in rats. The pulmonary pathological changes in each rat group were evaluated by H&E staining. The black arrow indicates changes in the alveoli. (A) The lung tissue structure in the Sham group was normal. (B) The GIRI group exhibited pulmonary alveolar wall edema with inflammatory cell infiltration. (C) The alveolar wall was relatively intact in the GIRI + GLD group. (D) The alveolar wall was largely intact with few inflammatory cell infiltrations in the GIRI + GLP group. Scale bar, 200 μm. (E–F) Western blot (E, F) and quantitative real-time PCR (qRT-PCR) (G) were used to assess the expression levels of apoptosis indicators, including Bax, Bcl-2, FAS, and FASL, in lung tissue. *P < 0.05 vs. Sham group; #P < 0.05 vs. GIRI group. GIRI, gut ischemia-reperfusion injury; GLD, gut lymphatic drainage; GLP, gut lymph purification.

We subsequently examined the impact of GLP on GIRI-induced ALI. Microscopic evaluation of hematoxylin and eosin-stained lung tissue sections revealed that the alveolar tissue structure was normal in the Sham group. Conversely, the alveolar structure was significantly damaged in the GIRI group. Notably, compared to the model or GIRI + GLD group, the lung tissue lesions were significantly improved in the GIRI + GLP group (Fig. 2 A–D). We also investigated the level of apoptosis in lung tissue. Western blot analysis revealed that, compared to the Sham group, the expression of apoptosis-related genes, including Bax, FAS, and FASL, was significantly increased in the GIRI group, while the expression of Bcl-2 was significantly reduced. Importantly, compared to the GIRI group, the expression levels of Bax, FAS, and FASL were significantly decreased, and the expression of Bcl-2 was significantly increased in both the GIRI + GLD and GIRI + GLP groups. Moreover, the GIRI + GLP group exhibited a more significant decrease in Bax, FAS, and FASL and a more significant increase in Bcl-2 compared to the GIRI + GLD group (Fig. 2E and F). The qRT-PCR results were consistent with those of the Western blot analysis, except for the absence of a significant difference in Bcl-2 expression among the lung tissues of each group (Fig. 2G).

### 3.3 GLP reduces DAMPs and increases anti-inflammatory factors in rats with ALI

**Fig. 3:**
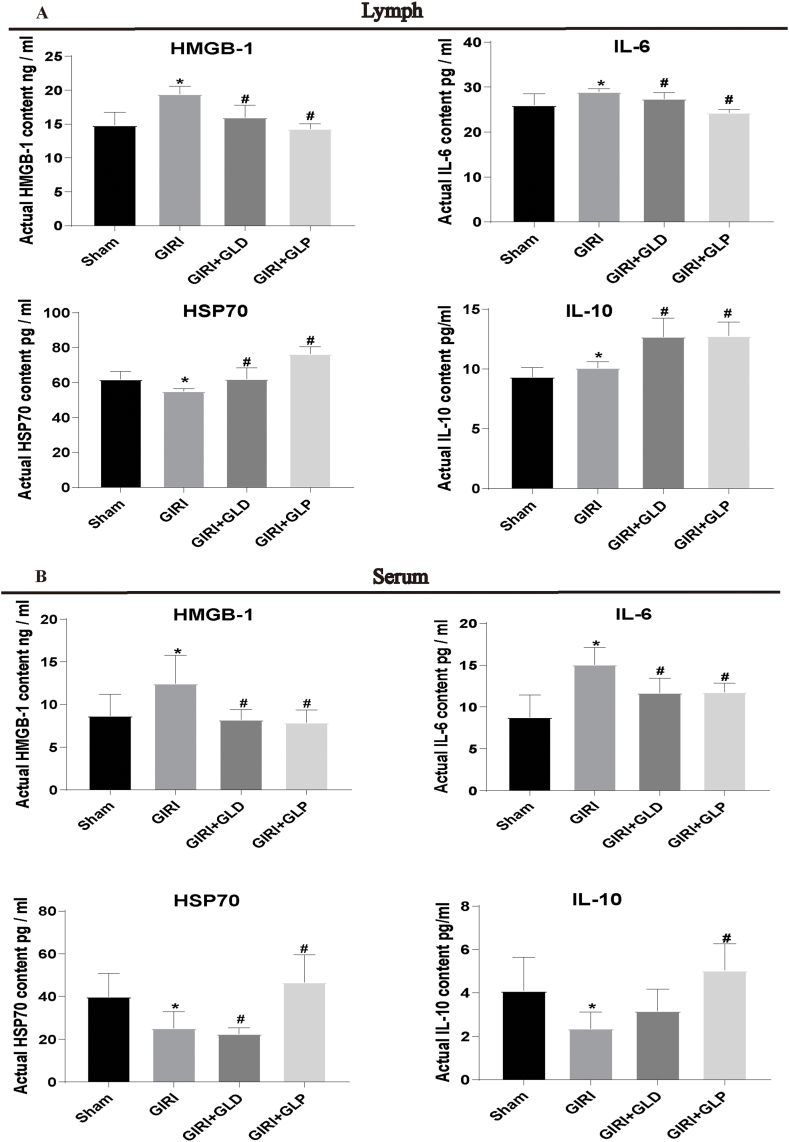
GLP reduced danger-associated molecular patterns (DAMPs) and increased anti-inflammatory factors in rats with ALI. Enzyme linked immunosorbent assay (ELISA) was performed to measure the levels of high mobility group box 1 (HMGB-1), interleukin- 6 (IL-6), interleukin-10 (IL-10), and heat shock protein 70 (HSP70) in gut lymph fluid (A) and serum (B) of rats. *P < 0.05 vs. Sham group; #P < 0.05 vs. GIRI group. HMGB-1, high mobility group box 1; HSP70, heat shock protein 70; IL-6, interleukin 6; IL-10, interleukin 10.

To investigate the potential anti-inflammatory effects of GLP during GIRI-induced ALI, we detected the DAMPs levels and anti-inflammatory factors in gut lymph fluid and serum of rats with ALI. Using ELISA, we observed a significant increase in the levels of pro-inflammatory mediators, such as high mobility group box 1 (HMGB-1) and interleukin- 6 (IL-6), in the GIRI group compared to the Sham group. Conversely, the levels of anti-inflammatory mediators, including interleukin-10 (IL-10) and heat shock protein 70 (HSP70), decreased in the GIRI group. Importantly, both GLD and GLP significantly reduced the levels of HMGB-1 and IL-6 while increasing the levels of HSP70 and IL-10. Furthermore, GIRI + GLP group exhibited higher levels of anti-inflammatory factors (HSP70 and IL-10) than the GIRI + GLD group (Fig. 3A and B). These findings suggest that GLP may attenuate inflammation during GIRI-induced ALI by modulating the levels of DAMPs and inflammatory factors.

### 3.4 GLP inhibits the apoptosis of mononuclear phagocytes and promotes the secretion of IL-10

**Fig. 4:**
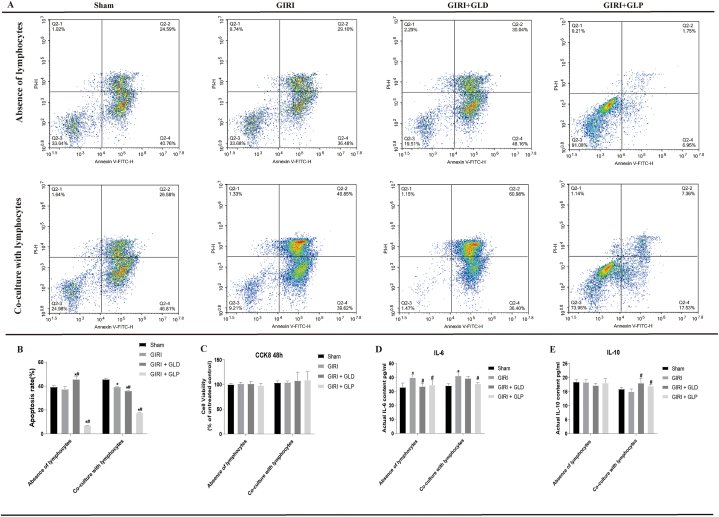
GLP inhibited the apoptosis of mononuclear phagocytes and promoted the secretion of IL-10. (A). Cell flow cytometry chart. (B). Histogram of apoptosis results. (C). Histogram of proliferation. (D, E). The levels of IL-6 and IL-10. *P < 0.05 vs. Sham group; #P < 0.05 vs. GIRI group. GIRI, gut ischemia-reperfusion injury; GLD, gut lymphatic drainage; GLP, gut lymph purification; IL-6, interleukin 6; IL-10, interleukin 10.

To explore the role of mononuclear phagocytes in the anti-inflammatory effect of GLP, we evaluated their apoptosis rates. Compared with the GIRI group, the apoptosis of mononuclear phagocytes in both GIRI + GLD and GIRI + GLP groups was significantly inhibited. GIRI + GLP group exhibited a more significant effect than the GIRI + GLD group (Fig. 4A and B). However, the CCK8 assay revealed no significant difference in cell viability between the groups (Fig. 4C). We also detected the levels of IL-6 and IL-10 in the supernatant of mononuclear phagocytes using ELISA. Our results showed that compared with the Sham group, the IL-6 levels were significantly increased in the GIRI group, while the levels of IL-10 did not change significantly. Among the mononuclear phagocytes in each group with lymphocyte culture, the GIRI group showed a significant increase in IL-6 levels and no significant change in IL-10 levels compared to the Sham group. Both GIRI + GLD and GIRI + GLP groups exhibited a significant increase in IL-10 levels, but IL-6 levels did not change significantly (Fig. 4D and E) compared with the GIRI group. This shows that GLP may inhibit the apoptosis of mononuclear phagocytes and promote IL-10 secretion to exert its anti-inflammatory effects.

### 3.5 GLP enhances the antigen presentation ability of dendritic cells

**Fig. 5:**
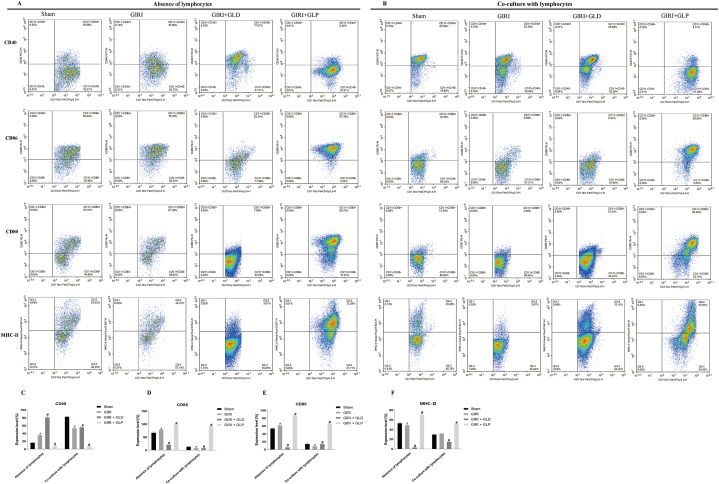
GLP enhanced the antigen presentation ability of dendritic cells. (A, B) Flow cytometry analysis of the expression levels of CD40, CD86, CD80, and MHC-II in Dendritic cells cocultured without lymphocytes (A) or with lymphocytes (B). (C, D, E, and F) Quantification of A and B. *\P < 0.05 vs. Sham group; #P < 0.05 vs. GIRI group. GIRI, gut ischemia-reperfusion injury; GLD, gut lymphatic drainage; GLP, gut lymph purification.

To evaluate the impact of GLP on dendritic cell antigen presentation ability, we detected CD40, CD86, and CD80 expression in dendritic cells co-cultured with or without lymphocytes (Fig. 5A and B). Without lymphocyte treatment, CD40, CD86, and CD80 expression levels were significantly upregulated in the GIRI group compared with the Sham group, while MHC-Ⅱ expression was significantly downregulated. In contrast, GIRI + GLP group had significantly downregulated CD40 expression and upregulated CD86, CD80, and MHC-Ⅱ expression compared with the GIRI group. In lymphocyte-treated dendritic cells, GIRI significantly downregulated CD40, CD86, and CD80 expression levels compared with the Sham group. In the GIRI + GLP group, CD40 expression was significantly downregulated, while CD86, CD80, and MHC-Ⅱ expression were significantly upregulated (Fig. 5C–F). These findings indicate that GLP promotes dendritic cell presentation ability, while GIRI inhibits it (Fig. 5).

### 3.6 GLP reduces AEC Ⅱ apoptosis

**Fig. 6:**
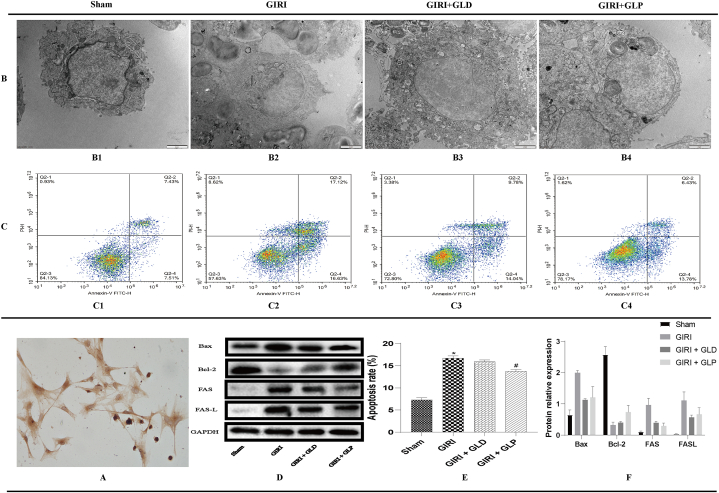
GLP reduced AEC Ⅱ apoptosis. (A). SP-A expression was detected by immunohistochemistry. (B). A transmission electron microscope was used to observe AEC Ⅱ microstructure in different groups (B1 = Sham, B2 = GIRI, B3 = GIRI + GLD, B4 = GIRI + GLP). (C). Flow cytometry was used to detect AEC Ⅱ apoptosis (C1 = Sham, C2 = GIRI, C3 = GIRI + GLD, C4 = GIRI + GLP). (D, E, and F) Western blot analysis of AEC Ⅱ apoptosis-related gene expression level. *P < 0.05 vs. Sham group; #P < 0.05 vs. GIRI group. GLP, gut lymph purification; GIRI, gut ischemia-reperfusion injury; AEC Ⅱ, alveolar epithelial type Ⅱ cell.

Next, we investigated if GLP protects AEC II from apoptosis by examining their identification and microstructure. A large amount of surfactant protein A (SP-A) was detected in primary AEC Ⅱ cells, confirming their identity (Fig. 6A). TEM revealed damaged organelle structure with vacuoles and reduced microvilli in the GIRI, while both GIRI + GLD and GIRI + GLP groups showed significant improvement in organelle structure with increased plate-shaped bodies (Fig. 6B). Flow cytometry showed increased apoptosis rate in the GIRI group and reduced rate in the GIRI + GLP group (Fig. 6C). Western blot analysis showed increased expression of Bax, FAS, and FASL and decreased expression of Bcl-2 in the GIRI group. Compared with the GIRI group, the expression levels of Bax, FAS, and FASL were reduced in both GIRI + GLD and GIRI + GLP groups, and Bcl-2 expression was significantly increased (Fig. 6D–F). This shows that GLP appears to have a protective effect against AEC II apoptosis in ALI induced by GIRI.

## 4 Discussion

This study showed that the use of GLP, combined with oXiris biofilm, alleviates ALI induced by GIRI in rats. This beneficial effect inhibits inflammation and cell apoptosis by blocking the interaction between DAMP in the intestinal lymphatic lung pathway and monocytes.

Mild injury to the intestinal mucosal epithelium occurs after 30 min of gut ischemia. With prolonged ischemic time, the intestinal mucosal cells become necrotic and release endogenous DAMPs [26], which can directly damage the intestinal mucosal epithelium cells, increase intestinal wall permeability, and promote the release of additional DAMPs from intestinal mucosal cells [27]. The translocated DAMPs enter the systemic circulation and activate the innate immune system in remote organs, resulting in the production of many stimulating mediators and cytokines, leading to a systemic inflammatory response [28].

**Fig. 7:**
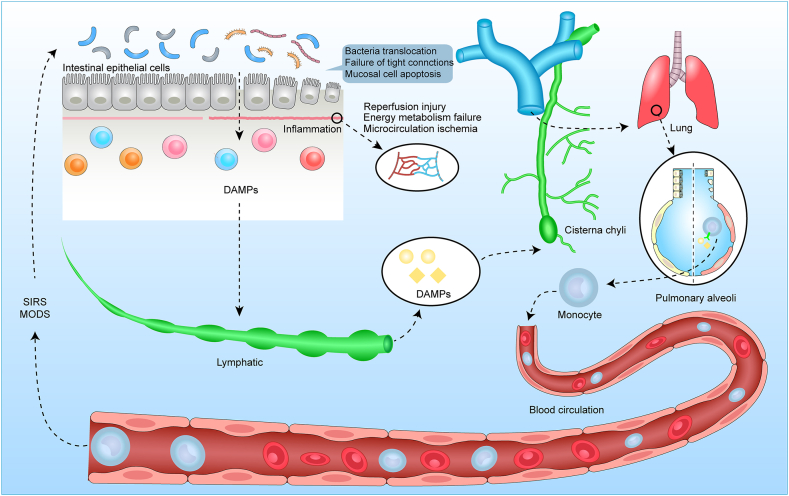
The pathophysiological mechanism of the gut lymph pathway that leads to the development of ALI and MODS. Organ hypoperfusion, ischemia-reperfusion injury (IRI), and intestinal nutrient deficiency promote intestinal injury through oxidative stress-mediated mechanisms. This results in the apoptosis of intestinal mucosal cells, destruction of tight junctions, and increased intestinal permeability. Intestinal bacteria and endotoxin cross the mucosal barrier and interact with the intestinal immune system, leading to proinflammatory reactions that worsen intestinal barrier dysfunction (the first vicious cycle). Due to intestinal mucosal injury, harmful biological molecules (DAMPs) are released into mesenteric lymphatic vessels, carried to the lungs, and then enter systemic circulation. These substances are recognized by cells carrying pattern recognition receptors (PRR) in the innate immune system, promoting proinflammatory pathways and leading to systemic inflammatory response syndrome (SIRS) and multiple organ dysfunction (MODS). The systemic inflammatory reaction further aggravates intestinal barrier damage, forming a second, potentially fatal vicious cycle. SIRS, systemic inflammatory response syndrome; MODS, multiple organ dysfunction syndrome; ARDS, acute respiratory distress syndrome; DAMPs, damage-associated molecular patterns.

The release of DAMPs following GIRI mainly involves the gut lymph pathway. Gut-derived factors carried in the gut lymph, rather than the portal blood, caused traumatic-hemorrhagic shock-induced lung injury, neutrophil activation, and endothelial cell activation and injury [29]. After GIRI, DAMPs translocate into the gut lymph fluid and then enter the systemic circulation, where they combine with Toll-like receptor 4 (TLR4) on peripheral mononuclear phagocytes to produce many inflammatory factors that target remote organs. These gut-derived DAMPs reach the lungs and systemic circulation via the gut lymph pathway, leading to the development of ALI and MODS (Fig. 7) [3].

**Fig. 8:**
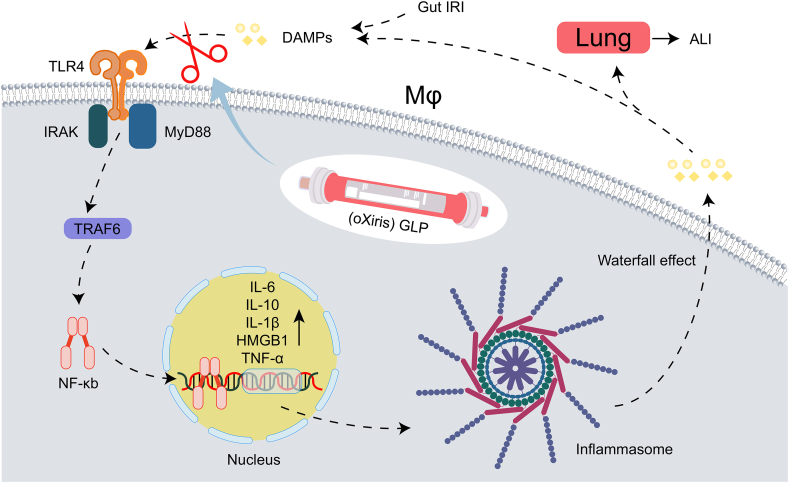
The oXiris-GLP therapeutic system interrupts the binding of DAMPs with TLR4 by clearing DAMPs from gut lymph. Gut IRI, gut ischemia-reperfusion injury; OXiris GLP, oXiris gut lymph purification; DAMPs: damage-associated molecular patterns; TLR4, Toll-like receptor 4; Inflammasome: inflammatory body; Mφ, mononuclear macrophages; IRAK, IL-1 receptor related kinase; MyD88, myeloid differentiation factor 88; TRAF6, TNF receptor related factor 6; NF-kB, nuclear factor kB; ALI, acute lung injury.

Current research on gut lymph intervention in sepsis has primarily focused on GLD and gut lymph ligation, both of which involve blocking the gut lymph system to prevent the spread of GIRI-induced damage to distant organs [19,30]. The gut lymph system supplements the systemic blood circulation system, playing a critical role in maintaining body fluid balance and nutrient metabolism. However, GLD and gut lymph ligation are anti-physiological treatment techniques that are difficult to apply in clinical practice. However, the oXiris-GLP therapeutic system offers a more physiological approach to addressing the link between DAMPs and mononuclear phagocytes. This system uses a lymphatic drainage catheter to establish an extracorporeal lymphatic circulation pathway that drains gut lymph fluid from the body, purifies it using the GLP system, and then infusion back into the body (Fig. 8). This approach meets the physiological requirements of critically ill patients and offers a promising alternative to existing gut lymph interventions.

Based on our results, GLP therapy for GIRI-induced ALI had significant effects on both acroscopic (lung tissue) and microscopic (AEC Ⅱ) aspects of the lungs, significantly inhibiting lung and cell apoptosis. Both GLD and GLP reduced DAMPs (HMGB-1, IL-6) in gut lymph fluid and peripheral blood, leading to reduce the triggering signal cascade by binding to TLR4 and induce activation of the nuclear factor kappa B pathway, which reduces inflammation. GLP significantly increased anti-inflammatory factors (HSP70 and IL-10) and reduced the apoptotic rate of mononuclear phagocytes, regardless of whether they were co-cultured with lymphocytes. GLP plays a two-way regulatory role in the innate immune response of the body, primarily by inhibiting the apoptosis of mononuclear phagocytes, suppressing the production of cytokines, and enhancing DC antigen presentation.

The role of gut lymph-derived DAMPs in the development of ALI following GIRI is well-established, but effective treatment options remain limited. Our team used a GLP technique based on oXiris membrane filtration that shows promise in addressing this issue by removing gut lymph-derived DAMPs in patients or rats with ALI caused by GIRI.

Despite the widespread use of continuous blood purification therapy in critically ill patients with sepsis-related complications, such as ALI and MODS, its poor therapeutic effects and adverse effects have prompted the exploration of extra measures. In response, we developed the oXiris-GLP system and explored its effectiveness in a rat model of ALI caused by GIRI. Our study provides basic medical evidence for the research and development of the ALI-GLP treatment system and lays the foundation for GLP therapy in the treatment of sepsis-related complications.

## 5 Conclusions

Our study found that oXiris-GLP effectively blocks the interaction between gut lymph-derived DAMPs and mononuclear phagocytes, which reduces inflammation and cell apoptosis, leading to a significant reduction in ALI induced by GIRI.

## Ethics declarations

This study was reviewed and approved by the Ethics Committee of Experimental Animals and Use of Laboratory Animals of Zunyi Medical University, with the approval number: LUN Review [2021] 2–049.

## Consent to publish

All authors have read and approved the manuscript and agree to submit it for consideration for publication in the journal. We confirm that we have read the journal's position on issues involved in ethical publication and affirm that this report is consistent with those guidelines.

## Availability of data and materials

The data that support the findings of this study are available from the Dryad Digital Repository at https://doi.org/10.5061/dryad.djh9w0w4j and are publicly available.

## Disclosure of conflicts of interest

The authors have declared that no competing interests exist.

## Funding

This study was supported by the Science and Technology Plan of Guizhou Province in 2020 (Foundation of Guizhou Science and Technology Cooperation [2020]1Z061). The funders had no role in study design, data collection and analysis, decision to publish, or preparation of the manuscript.

## Data availability statement

The data that support the findings of this study are available from the Dryad Digital Repository at https://doi.org/10.5061/dryad.djh9w0w4j and are publicly available and the accession number is my email named by zhangwei_hxicu@163.com.

## CRediT authorship contribution statement

**Wei Zhang:** Writing – review & editing, Writing – original draft, Visualization, Validation, Supervision, Software, Resources, Project administration, Methodology, Investigation, Funding acquisition, Formal analysis, Conceptualization. **Can Jin:** Writing – original draft, Visualization, Validation, Software, Resources, Methodology, Investigation, Formal analysis, Data curation. **Shucheng Zhang:** Writing – original draft, Visualization, Validation, Software, Methodology, Investigation, Formal analysis, Data curation. **Linlin Wu:** Visualization, Validation, Resources, Methodology, Investigation, Data curation. **Bohan Li:** Visualization, Validation, Software, Methodology, Investigation, Formal analysis, Data curation. **Meimei Shi:** Writing – review & editing, Validation, Supervision, Project administration, Methodology, Investigation, Conceptualization.

## Declaration of competing interest

The authors declare that they have no known competing financial interests or personal relationships that could have appeared to influence the work reported in this paper.

## Abbreviations List

MODS: multiple organ dysfunction syndrome
DAMPs: danger-associated molecular patterns
GIRI: gut ischemia reperfusion injury
ALI: acute lung injury
CBP: continuous blood purification
GLP: gut lymph purification
GLD: gut lymph drainage
HE: hematoxylin-eosin
AEC Ⅱ: type II alveolar epithelial cells
HMGB-1: high mobility group box 1
IL-6: interleukin- 6
IL-10: interleukin-10
HSP 70: heat shock protein 70
SMA: superior mesenteric artery
PBS: phosphate buffered solution
PMA: Phorbol-12-myristate-13-acetate
ELISA: enzyme-linked immunosorbent assay
WB: Western Blot
PMSF: phenylmethylsulphonyl fluoride
EP: Eppendorf
BCA: bicinchoninic acid
qRT-PCR: quantitative real-time polymerase chain reaction
miRNA: micro ribonucleic acid
cDNA: complementary deoxyribonucleic acid
CCK8: cell counting kit-8
TEM: transmission electron microscopy
OI: oxygenation index
NO: nitric oxide
PLA2: phospholipase A2
TNF-α: tumor necrosis factor-α
SP-A: surfactant protein A
TLR4: Toll-like receptor 4
NF-κB: nuclear factor kappa B
